# Using an incomplete block design to allocate lines to environments improves sparse genome‐based prediction in plant breeding

**DOI:** 10.1002/tpg2.20194

**Published:** 2022-02-16

**Authors:** Osval Antonio Montesinos‐Lopez, Abelardo Montesinos‐Lopez, Ricardo Acosta, Rajeev K. Varshney, Alison Bentley, Jose Crossa

**Affiliations:** ^1^ Facultad de Telemática Univ. de Colima Colima Colima 28040 México; ^2^ Centro Universitario de Ciencias Exactas e Ingenierías (CUCEI) Univ. de Guadalajara Guadalajara Jalisco 44430 México; ^3^ International Crops Research Institute for the Semi‐Arid Tropics (ICRISAT) Hyderabad India; ^4^ State Agricultural Biotechnology Centre, Centre for Crop and Food Innovation, Food Futures Institute Murdoch Univ. Murdoch Australia; ^5^ International Maize and Wheat Improvement Center (CIMMYT) Km 45, Carretera México‐Veracruz, CP 52640 Edo. de México México; ^6^ Colegio de Postgraduados Montecillos Edo. de México CP, 56230 México

## Abstract

Genomic selection (GS) is a predictive methodology that trains statistical machine‐learning models with a reference population that is used to perform genome‐enabled predictions of new lines. In plant breeding, it has the potential to increase the speed and reduce the cost of selection. However, to optimize resources, sparse testing methods have been proposed. A common approach is to guarantee a proportion of nonoverlapping and overlapping lines allocated randomly in locations, that is, lines appearing in some locations but not in all. In this study we propose using incomplete block designs (IBD), principally, for the allocation of lines to locations in such a way that not all lines are observed in all locations. We compare this allocation with a random allocation of lines to locations guaranteeing that the lines are allocated to the same number of locations as under the IBD design. We implemented this benchmarking on several crop data sets under the Bayesian genomic best linear unbiased predictor (GBLUP) model, finding that allocation under the principle of IBD outperformed random allocation by between 1.4% and 26.5% across locations, traits, and data sets in terms of mean square error. Although a wide range of performance improvements were observed, our results provide evidence that using IBD for the allocation of lines to locations can help improve predictive performance compared with random allocation. This has the potential to be applied to large‐scale plant breeding programs.

## Core Ideas

- Incomplete block design (IBD) principle is applied in sparse field testing.
- Genome‐based sparse testing from IBD concept is proposed.
- Sparse testing across environments for genome‐based  prediction is optimized.
- Genome‐based prediction sparse testing with IBD includes G×E interaction.

**Abbreviations**
Bed5IR: bed planting with five irrigations
BLUE: best linear unbiased estimate
DTHD: days to heading
DTMT: days to maturity
EHT: early heat
EYT: elite wheat yield trial
Flat5IR: flat planting with five irrigations
FlatDrip: flat planting with drip irrigation
G×E: genotype × environment (or location)
GBLUP: genomic best linear unbiased predictor
GE: model considers the G×E interaction
GS: genomic selection
GY: grain yield
IBD: incomplete block design
LHT: late heat
MSE: mean square error
MSE_IBD: MSE under IBD allocation
MSE_Random: MSE under random allocation and
NO_GE: model ignores the G×E interaction
NPP: number of pods per plant
PYPP: pod yield per plant
RE: relative efficiency
SNP: single‐nucleotide polymorphism
SYPP: seed yield per plant
YPH: yield per hectare.

## 1 INTRODUCTION

Genomic selection (GS) was proposed by Meuwissen et al. (2001) to exploit dense genome‐wide markers for predicting complex traits. It is a predictive methodology that trains a statistical machine‐learning model using a reference population (with phenotypic and genotypic information) to calculate predicted breeding or phenotypic values for new lines that were only genotyped. For this reason, GS allows candidate lines to be selected early in the selection process, and under a careful and efficient implementation, offers tremendous opportunities to improve rates of genetic gain in plant and animal breeding (Bhat et al., 2016; Crossa et al., 2017; Heffner et al., 2010; Zhong et al., 2009).

The objective of plant breeding is genetic improvement by producing new genotypes (lines) with improved productivity and quality. In many plant breeding programs at preliminary breeding stages, a majority of hybrids are generated by crossing doubled‐haploids lines (or lines developed using a pedigree scheme) to a tester from a complementary heterotic group. The test‐cross hybrids are evaluated in some locations (3–5), and subsequently, the best 10–15% of the lines within or across locations are selected to advance to further yield trials (Beyene et al., 2019). Effective selection decisions at the initial stage of yield testing (typically denoted Stage 1) are critical for the advancement of lines with the greatest potential to perform in the resource‐intensive multilocation, multitester testing stages (typically denoted Stage 2; Atanda, et al., 2021). However, phenotypic selection in Stage 1 material is not completely effective because of the presence of only one tester for test‐cross hybrid evaluation in a few locations, which do not guarantee a representative sample of the target population of locations (Endelman et al., 2014).

Multilocation trials are key elements in successful breeding programs, permitting the evaluation of promising candidate genotypes under different locational conditions. As such, it is possible to identify stable genotypes or genotypes with specific adaptation by modeling the genotype × environment (or location) (G×E) interaction. However, the ideal implementation where all genotypes are observed in each location requires extensive field testing and considerable resource allocation (Smith et al., 2015a, 2015b).

Experimental designs are powerful tools that have historically been used in breeding programs to increase the precision or reduce the cost of generating parameter estimates in field trials. Popular experimental designs in plant breeding include randomized block designs, incomplete blocks designs (IBD), row–column designs, or α designs (see Bailey [2008], John and Williams [1995], and Patterson and Williams [1976], for examples). For early generation testing, both the *p*‐rep design developed by Cullis et al. (2006) and *p*‐rep with augmented designs (where only checks are repeated developed by Williams et al. [2011]) are popular.

Sparse testing is a technique where not all lines are observed in all locations, with lines allocated to locations using a sparse testing design. For example, cross‐validation CV2 evaluates the prediction accuracy of models when some genotypes have been evaluated in some locations but not in others and can be used for building sparse testing designs. However, it is also possible to use many traditional experimental designs to allocate treatment to plots or blocks and thus build a sparse testing design. This reshapes the original multilocation breeding trial system into one where all lines are not replicated in all locations, as high costs and factors like seed, land, and water availability might impede the implementation of replicated trials.

In this study we investigate the use of IBDs to more efficiently allocate lines to locations in order to enable sparse genomic prediction. We also compare predictive performance from the allocation of lines to locations using IBD against the conventional random allocation using three crop species—wheat (*Triticum aestivum* L.), groundnut (*Arachis hypogaea* L.), and maize (*Zea mays* L.)—each including different traits data. This comparison of prediction accuracy uses mean squared error (MSE) of prediction of the IBD and random allocation implemented under the popular Bayesian genomic best linear unbiased predictor (GBLUP) model, which was used for comparison, as it is the most widely used model in genome‐enabled prediction. The resulting predictions under the two methods were also compared in the absence (NO_GE) and presence (GE) of G×E interactions.

## 2 MATERIALS AND METHODS

Data sets used for the benchmarking of the two allocation methods are described below.

**Core Ideas**

- Incomplete block design (IBD) principle is applied in sparse field testing.
- Genome‐based sparse testing from IBD concept is proposed.
- Sparse testing across environments for genome‐based  prediction is optimized.
- Genome‐based prediction sparse testing with IBD includes G×E interaction.

### 2.1 Data Sets 1 and 2. Elite wheat yield trial years 2013–2014 and 2016–2017

Two data sets from the Global Wheat Program at the International Maize and Wheat Improvement Center (CIMMYT) were used. They consisted of performance data from elite wheat yield trials (EYTs) established in four different cropping seasons with four locations in each. The lines involved in this study correspond to years 2013–2014 (Data Set 1) and to 2016–2017 (Data Set 2). The EYT Data Set 1 and Data Set 2 contain 766 lines and 980 lines, respectively. In both data sets, an experimental alpha‐lattice design was used where the lines were sown in 39 trials, each covering 28 lines and two checks in six blocks with three replications. In these data sets, several traits were available for the selection of locations and lines. In this study, we included four traits that were measured for each line in each location: days to heading (DTHD, number of days from germination to 50% spike emergence); days to maturity (DTMT, number of days from germination to 50% physiological maturity or the loss of the green color in 50% of the spikes); plant height in cm; and grain yield (GY in tons by hectare). Full details of the experimental design and computation of best linear unbiased estimates (BLUEs) can be found in Juliana et al. (2018). For EYT Data Set 1, the selected locations were bed planting with five irrigations (Bed5IR), flat planting with five irrigations (Flat5IR), early heat (EHT), and late heat (LHT). For EYT Data Set 2, the locations were Bed5IR, EHT, Flat5IR, and flat planting with drip irrigation (FlatDrip).

Genome‐wide markers for the 1,746 (766 + 980) lines in the two data sets were obtained using genotyping‐by‐sequencing (Elshire et al., 2011; Poland et al., 2012) at Kansas State University using an Illumina HiSeq2500. After filtering, 2,038 markers remained from an initial set of 34,900 markers. The imputation of missing marker data was carried out using LinkImpute (Money et al., 2015) and implemented in TASSEL v5 (Bradbury et al., 2007). Lines that had >50% missing data were removed, thus providing a total of 1,506 lines for this study (766 lines in the first data set and 980 lines in the second data set). A high level of relatedness by pedigree or kinship between lines is expected within a year of testing and across years of testing because of the nature of the lines under study.

### 2.2 Data Set 3. Groundnut

The phenotypic data set reported by Pandey et al. (2020) includes information on the phenotypic performance of 318 groundnut lines for various traits in four locations. We assessed genomic‐enabled predictions for the following four traits: pods per plant (NPP), pod yield per plant (PYPP) measured in grams, seed yield per plant (SYPP) in grams, and yield per hectare (YPH) in kilograms. The locations are denoted as Location 1 (ENV1: Aliyarnagar_Rainy 2015), Location 2 (ENV2: Jalgoan_Rainy 2015), Location 3 (ENV3: ICRISAT_Rainy 2015), and Location 4 (ENV4: ICRISAT Post‐Rainy 2015). The data set is balanced, giving a total of 1,272 assessments with each line included once in each location. Marker data were available for all lines, and 8,268 single‐nucleotide polymorphism (SNP) markers remained after quality control (with each marker coded with 0, 1, or 2).

### 2.3 Data Set 4. Wheat data

This data set was first used by Crossa et al. (2010) and Cuevas et al. (2016, 2017, 2019) and is comprised of 599 wheat lines from the CIMMYT Global Wheat Program evaluated in four international locations representing four basic agroclimatic regions (mega‐locations). Here, we considered GY data available for the lines evaluated in each of the four mega‐locations. The 599 wheat lines were genotyped using 1,447 diversity array technology markers generated by Triticarte Pty. Ltd.

### 2.4 Data Set 5. Maize data

This maize data set was included in Souza et al. (2017), originating from Universidad Sao Paulo and consisting of 722 maize hybrids obtained by crossing 49 inbred lines. The hybrids were evaluated in four locations (E1–E4) in Piracicaba and Anhumas, São Paulo, Brazil, in 2016 to yield a total of 2,888 observations (722 hybrids × 4 locations). The hybrids were evaluated using an augmented block design with two commercial hybrids as checks to correct for microlocational variation. At each site, two levels of nitrogen (N) fertilization were used: ideal N conditions (plots received 100 kg ha^−1^ of N [30 kg ha^−1^ at sowing and 70 kg ha^−1^ in a coverage application] at the V8 plant stage) and low N (plots received 30 kg ha^−1^ of N at sowing). The parental lines were genotyped with an Affymetrix Axiom Maize Genotyping Array (Unterseer et al., 2014) of 616 K SNPs. Markers with minor allele frequency of 0.05 were removed. After applying quality control, 54,113 SNPs were available for predictions.

### 2.5 Bayesian GBLUP model

The Bayesian GBLUP model is represented by the following equation:

(1) $$Y_{\mathrm{ij}}=\;\mu +L_{i}+g_{j}+gL_{\mathrm{ij}}+\varepsilon_{\mathrm{ij}}$$

where *L_i_* is the fixed effect of locations; *g_j_*, where *j* = 1,…,*J*, is the random effect of lines; *gL_ij_* is the random effect of location−line interaction; and ε*_ij_* is random error components in the model assumed to be independent normal random variables with mean 0 and variance σ^2^. Furthermore, it is assumed that $g={\left(g_{1},\text{…},g_{J}\right)}^{T}∼N_{J}\left(0,\sigma_{g}^{2}G\right)$and $\mathrm{gL}={\left(gL_{11},\text{…},gL_{1J},\text{…},\;gL_{IJ}\right)}^{T}∼N_{IJ}\left[0,\sigma_{\mathrm{gL}}^{2}\left(I⊗G\right)\right]$, where **G** is the genomic relationship matrix as computed by VanRaden (2008), ⊗ denotes the Kronecker product, and **I** is the identity matrix of size *I*. The implementation of this model was carried out in the BGLR library of Pérez and de los Campos (2014). It is important to point out that this model (Equation 1) contains G×E interaction but was also implemented without G×E interaction (NO_GE), that is, the model without the fourth component on the right side of Equation 1.

Under both types of allocation methods, IBD and random allocation, we use the notation *J* as the number of lines, *k* as the number of lines per location, *I* as the number of locations, and *r* as the number of replications of each line *j* in the entire design. It should be noted that in IBDs, *k* will be less than *J*, since not all of the lines in each location can be assigned. An equal number of entry replication is the best way to ensure minimum variance when making all possible pairwise comparisons. Therefore, since *r_i_* = *r* for all lines, the total number of observations in the experiment is *N*, where *N* = *J*(*r*) = *I*(*k*).

### 2.6 Allocation of lines to locations using the IBD method

A balanced IBD design is where all pairs of lines occur together within a location an equal number of times (λ). In general, we will specify λ*_jj_* as the number of times line *j* occurs with *j′′* in a location. To generate this sparse allocation of lines to locations, we can use the function find.BIB() using the R package crossdes. For example, suppose there were *J* = 12 lines and *I* = 4 locations, this means that we need 48 plots to allocate the 12 lines to the four locations. However, assume that we will use an IBD and a training set equal in size to *N_TRN* = 36 (75%) of the total plots required under a randomize complete block design. Therefore, the number of lines by locations can be obtained by solving (*kI* = N_TRN) for *k*, which results in *k* = N_TRN/*I*. This means that *k* = 36/4 = 9 lines per location. Then, the corresponding elements for the training set can be obtained with the function find.BIB(12, 4, 9) using the package crossdes. The numbers used in the function find.BIB() denote the lines, the locations, and the lines per locations, respectively. Finally, the lines tested in each location that correspond to the training set are shown in Table 1.

**TABLE 1:** Allocation of *J* = 12 lines to *I* = 4 locations under the incomplete block design method. This information allocated represents the training set (75%) and the size of the location, which is equal to nine, and each line is repeated *r* = *b*(*k*)/*J* = 36/12 = 3 times

**Locations**	**1**	**2**	**3**	**4**	**5**	**6**	**7**	**8**	**9**
Env1	G1	G3	G4	G5	G6	G7	G9	G11	G12
Env2	G1	G2	G3	G5	G7	G8	G9	G10	G11
Env3	G2	G3	G4	G5	G6	G8	G10	G11	G12
Env4	G1	G2	G4	G6	G7	G8	G9	G10	G12

Based on Table 1, each line is present in three locations and missing in one. All the lines shown in Table 1 correspond to the training set, while those not allocated in each location form the testing set. For example, in Location 1, the test set includes lines G2, G8, and G10; in Location 2, the test set is comprised of lines G4, G6, and G12; in Location 3, the test set has lines G1, G7, and G9; and in Location 4, the test set is comprised of lines G3, G5, and G11. It is important to highlight that the function does not always guarantee a balanced IBD, and for this reason, we generally use the IBD method to guarantee a balanced or a partially balanced IBD (Sailer, 2013).

### 2.7 Random allocation of lines to locations

Starting from a balanced data set with *J* lines and *I* locations, the conformation of the random allocation of lines to locations was done in such a way that approximately each line will be repeated in *r* out of *I* locations, and all locations will be of the same size (*k*). The algorithm of this random allocation is as follows:

1. First, we compute $k=\frac{Jr}{I}$ (least integer greater than or equal to $\frac{Jr}{I}$). Then *k* lines out of *J* lines are randomly allocated to the first location.
2. Then for the second location, *k* out of the *J* lines were again randomly allocated.
3. This process is repeated until the *I*th location is completed, with the caveat that the lines allocated to a particular location are only present in less than or equal to *r* locations, ideally in exactly *r* locations. The lines that do not satisfy this restriction are not candidates for allocation to a particular location.

### 2.8 Cross‐validation strategy

To evaluate and compare the predictive performance of the IBD and random allocations, we used cross‐validation with 10 random partitions and 50% of the data for training and 50% for testing. The average MSE was computed with the 10 random partitions and this metric was used to assess the predictive performance in each data set. For each location in each data set, the predictive performance in terms of MSE was computed as the average of the 10 MSEs in the 10 random partitions. Across locations, the MSE in each partition was computed between averages of true and predicted phenotypic values over locations; subsequently, the average of the MSEs of the 10 partitions was reported as prediction performance in each data set. It must be highlighted that 50% of the data was used for training–testing in each partition since each of the five data sets under study included four locations. Therefore, under both types of allocations, we guaranteed that each line was replicated exactly two times (in two locations). Those lines allocated under the IBD and random allocations were used as training and the remaining were used as testing sets. To compare the predictive performance between the IBD and random allocation, we computed the relative efficiency (RE) as follows:

$$\mathrm{RE}=\frac{\mathrm{MSE}\_\mathrm{Random}}{\mathrm{MSE}\_\mathrm{IBD}}$$

where MSE_Random is the MSE under random allocation and MSE_IBD is the MSE under IBD allocation. The RE indicates how much more efficient (in percentage terms if the RE is multiplied by 100) the IBD allocation is in comparison with the random allocation; if the value of RE is >1 then the IBD allocation results in a smaller prediction error; however, if the RE is <1, the IBD allocation is less efficient (with more prediction error) than the random allocation. Relative efficiency is commonly used to make comparisons between randomized complete block designs and IBDs (Kuehl, 2001).

## 3 RESULTS

First, we provide a summary of the phenotypic values and variance components of each trait for each data set. The summary of each trait for all data sets is given in Table 2, where we can see that each trait has a different scale and varies significantly, as exemplified by its minimum and maximum values of each trait. We can also see that that the GY traits of the wheat and maize data sets are scaled for this reason, as they yielded values between −3.58 and 4.88. Likewise, we can appreciate that the mean and median are different for most of the traits except for YPH, PYPP, and NPP in the groundnut data set and height in Data Set 1 (EYT) and Data Set 2 (EYT). The difference between the mean and median was stronger, and for this reason, the data are more asymmetric for these traits.

**TABLE 2:** Summary of the phenotypic responses for each of the five data sets

**Data**	**Trait**	**Min**.	**1st quantile**	**Median**	**Mean**	**3rd quantile**	**Max**.
Data Set 1 (EYT)	DTHD (d)	52.00	68.00	79.33	77.22	85.33	112.00
	DTMT (d)	76.00	105.50	120.70	116.00	127.70	154.00
	GY	1.71	4.15	6.11	5.52	6.57	7.96
	Height	50.00	85.92	100.00	93.02	104.33	119.00
Data Set 2 (EYT)	DTHD	4.00	71.00	75.00	74.91	78.00	100.00
	DTMT	100.00	112.00	116.00	117.70	124.00	144.00
	GY	0.95	3.67	6.19	5.45	6.68	7.96
	Height	59.00	91.00	98.00	95.90	103.00	117.00
Groundnut	NPP	2.85	10.00	13.30	13.86	17.36	35.10
	PYPP (g)	1.94	6.71	9.10	9.59	11.82	29.03
	SYPP (g)	0.74	3.92	5.37	5.66	7.00	17.21
	YPH (kg)	356.80	995.10	1428.40	1556.30	2024.20	4864.50
Wheat	GY	−3.58	−0.63	0.02	0.00	0.63	4.88
Maize	GY	−2.35	−0.30	0.01	0.01	0.31	2.46

*Note*. DTHD, days to heading; DTMT, days to maturity; EYT, elite wheat yield trial; GY, grain yield; NPP, number of pods per plant; PYPP, pod yield per plant; SYPP, seed yield per plant; YPH, yield per hectare.

In Table 3, we can see the variance components of locations (L), genotypes (G), genotype × location (G×E) interaction, residual, R, and total (and its corresponding proportion of total variability) explained for each component in each trait of all the data sets. We can see (Table 3) that in Data Set 1 (EYT), the largest proportion of total variability was explained by the locations, while the second largest was for lines in traits DTHD, DTMT and Height. In the GY trait, the second largest was in the G×E and residual. In Data Set 2 (EYT), the largest proportion of variability was explained by locations in three out of the four traits, whereas in the DTHD trait, the largest proportion of variability was explained by the genotypes. However, in the groundnut data set, the largest proportion of variability was explained by the G×E and residual variance components. Conversely, in the wheat data set, the largest proportion of variability was explained by the residual and the second largest by the G×E variance component. Finally, in the maize data set, the largest proportion of variability was also explained by the G×E and residual terms (Table 3). In Appendix A, biplots for each trait of each data set show how similar and different the locations and cultivar under study are based on the site regression model (Crossa & Cornelius, 1997).

**TABLE 3:** Variance components for locations (*L*), genotypes (*G*), genotype × location (G×L), residual (*R*), and total. Total was computed as the sum of the variance components of all variance components for each trait. The proportion of the total variation (%) for each variance component is shown with its respective estimate

**Data**	**Trait**	***L***		***G***		**G×L**		***R***		**Total**	
		Estimate	%	Estimate	%	Estimate	%	Estimate	%	Estimate	%
Data Set 1 (EYT)	DTHD	**108.350**	**0.764**	22.285	0.157	5.568	0.039	5.569	0.039	141.772	1.0
	DTMT	**378.780**	**0.930**	18.365	0.045	5.109	0.013	5.109	0.013	407.363	1.0
	GY	**2.510**	**0.899**	0.055	0.020	0.113	0.040	0.113	0.040	2.790	1.0
	Height	**353.810**	**0.934**	9.638	0.025	7.758	0.020	7.752	0.020	378.958	1.0
Data Set 2 (EYT)	DTHD	3.903	0.122	**20.229**	**0.632**	3.935	0.123	3.937	0.123	32.004	1.0
	DTMT	**58.203**	**0.688**	20.811	0.246	2.778	0.033	2.779	0.033	84.572	1.0
	GY	**3.502**	**0.925**	0.064	0.017	0.111	0.029	0.110	0.029	3.787	1.0
	Height	**71.527**	**0.654**	6.257	0.057	15.780	0.144	15.749	0.144	109.312	1.0
Groundnut	NPP	5.438	0.169	8.680	0.269	**9.066**	**0.281**	**9.062**	**0.281**	32.246	1.0
	PYPP	3.853	0.215	3.234	0.181	**5.397**	**0.302**	**5.395**	**0.302**	17.879	1.0
	SYPP	1.307	0.191	1.342	0.196	**2.101**	**0.307**	**2.100**	**0.307**	6.849	1.0
	YPH	11,921	0.022	203,461	0.372	**165,384**	**0.303**	**165,499**	**0.303**	546,265.000	1.0
Wheat	GY	0.000	0.000	0.188	0.188	0.405	0.405	**0.407**	**0.407**	0.999	1.0
Maize	GY	0.000	0.000	0.109	0.377	**0.090**	**0.312**	**0.090**	**0.312**	0.290	1.0

*Note*. DTHD, days to heading; DTMT, days to maturity; EYT, elite wheat yield trial; GY, grain yield; NPP, number of pods per plant; PYPP, pod yield per plant; SYPP, seed yield per plant; YPH, yield per hectare. The variance components with the largest proportion of total variability explained are shown in bold.

### 3.1 Data Set 1 (EYT years 2013–2014)

First, we present the results including the G×E interaction of the prediction performance for each location. In Table 4 we can observe that the best predictions in terms of MSE were observed under the IBD allocation since REs in most cases were >1 for each of the traits. For trait DTHD, the REs observed were 1.013 (Bed5IR), 1.026 (EHT), 1.132 (Flat5IR), and 1.264 (LHT), which means that the IBD was more efficient than the random allocation by 1.3, 2.6, 13.2, and 26.4% in locations Bed5IR, EHT, Flat5IR, and LHT, respectively (Table 4). For trait DTMT, the REs were 1.030 (Bed5IR), 0.984 (EHT), 1.078 (Flat5IR), and 1.131 (LHT), which means that the IBD was more efficient than the random allocation by 3.0, 7.8, and 13.1% in locations Bed5IR, Flat5IR, and LHT, respectively (Table 4). For trait GY, the IBD was more efficient than the random allocation by only 2.0, 1.9, 0.1, and 1.1% in locations Bed5IR, EHT, Flat5IR, and LHT, respectively. While for trait height, the IBD outperformed the random allocation by only 2.1, 1.3, 2.3, and 4.1% in locations Bed5IR, EHT, Flat5IR, and LHT, respectively (Table 4).

**TABLE 4:** Data Set 1. Prediction performance in terms of mean square error (MSE) for each location for Data Set 1 (elite wheat yield trial years 2013–2014)

**Interaction**	**Trait**	**Location**	**MSE_IBD**	**SE_1**	**MSE_Random**	**SE_2**	**RE**
GE	DTHD	Bed5IR	17.803	0.365	18.038	0.349	1.013
		EHT	33.096	0.691	33.943	0.498	1.026
		Flat5IR	6.242	0.196	7.064	0.185	1.132
		LHT	4.548	0.131	5.748	0.172	1.264
	DTMT	Bed5IR	14.934	0.303	15.385	0.370	1.030
		EHT	31.285	0.602	30.799	0.385	0.984
		Flat5IR	5.225	0.119	5.635	0.115	1.078
		LHT	7.942	0.181	8.979	0.200	1.131
	GY	Bed5IR	0.163	0.003	0.167	0.003	1.020
		EHT	0.317	0.005	0.323	0.006	1.019
		Flat5IR	0.313	0.005	0.314	0.006	1.001
		LHT	0.140	0.004	0.142	0.003	1.011
	Height	Bed5IR	12.319	0.273	12.584	0.239	1.021
		EHT	23.691	0.496	23.988	0.545	1.013
		Flat5IR	13.662	0.214	13.976	0.234	1.023
		LHT	26.935	0.441	28.034	0.528	1.041
NO_GE	DTHD	Bed5IR	18.612	0.290	19.262	0.313	1.035
		EHT	35.028	0.814	36.103	0.397	1.031
		Flat5IR	8.551	0.252	9.298	0.188	1.087
		LHT	5.731	0.137	6.883	0.205	1.201
	DTMT	Bed5IR	14.322	0.247	15.429	0.346	1.077
		EHT	31.124	0.691	31.096	0.330	0.999
		Flat5IR	7.635	0.169	7.934	0.174	1.039
		LHT	8.485	0.143	9.720	0.187	1.145
	GY	Bed5IR	0.164	0.002	0.167	0.003	1.018
		EHT	0.339	0.006	0.341	0.006	1.006
		Flat5IR	0.310	0.005	0.310	0.007	1.001
		LHT	0.184	0.004	0.181	0.004	0.983
	Height	Bed5IR	12.271	0.286	12.686	0.241	1.034
		EHT	25.380	0.648	25.586	0.510	1.008
		Flat5IR	14.251	0.229	15.106	0.282	1.060
		LHT	27.339	0.540	29.020	0.550	1.061

*Note*. Bed5IR, bed planting with five irrigations; DTHD, days to heading; DTMT, days to maturity; EHT, early heat ; Flat5IR, flat planting with five irrigations; GE, model considers the genotype × location interaction; GY, grain yield; IBD, incomplete block design; LHT, late heat; MSE_IBD, MSE under IBD allocation; MSE_Random, MSE under random allocation; NO_GE, model ignores the genotype × location interaction; NPP, number of pods per plant; PYPP, pod yield per plant; RE, relative efficiency computed as the ratio of the MSE_IBD/MSE_Random; SE_1, SE of the MSE under the IBD allocation while ; SE_2, SE of the MSE under the random allocation; SYPP, seed yield per plant; YPH, yield per hectare.

Ignoring the G×E interaction (NO_GE), in Table 4 we can also observe that the IBD allocation outperformed the random allocation in each location since, in most cases, the REs were >1 for the four traits. For DTHD trait, the REs observed were 1.035, 1.031, 1.087, and 1.201 in locations Bed5IR, EHT, Flat5IR, and LHT, respectively. Therefore, in this trait, the IBD allocation outperformed the random allocation by 3.5 (Bed5IR), 3.1 (EHT), 8.7 (Flat5IR), and 20.1% (LHT) (Table 4). For the DTMT trait, the REs were 1.077 (Bed5IR), 0.999 (EHT), 1.039 (Flat5IR), and 1.145 (LHT), which means that the IBD outperformed the random allocation by 7.7 (Bed5IR), 3.9 (Flat5IR), and 14.5% (LHT). For the GY trait, the IBD outperformed the random allocation by 1.8, 0.6, and 0.1% in locations Bed5IR, EHT, and Flat5IR, respectively. While for height, the IBD was superior to the random allocation by only 3.4, 0.8, 6.0, and 6.1% in locations Bed5IR, EHT, Flat5IR, and LHT, respectively (Table 4).

**FIGURE 1:**
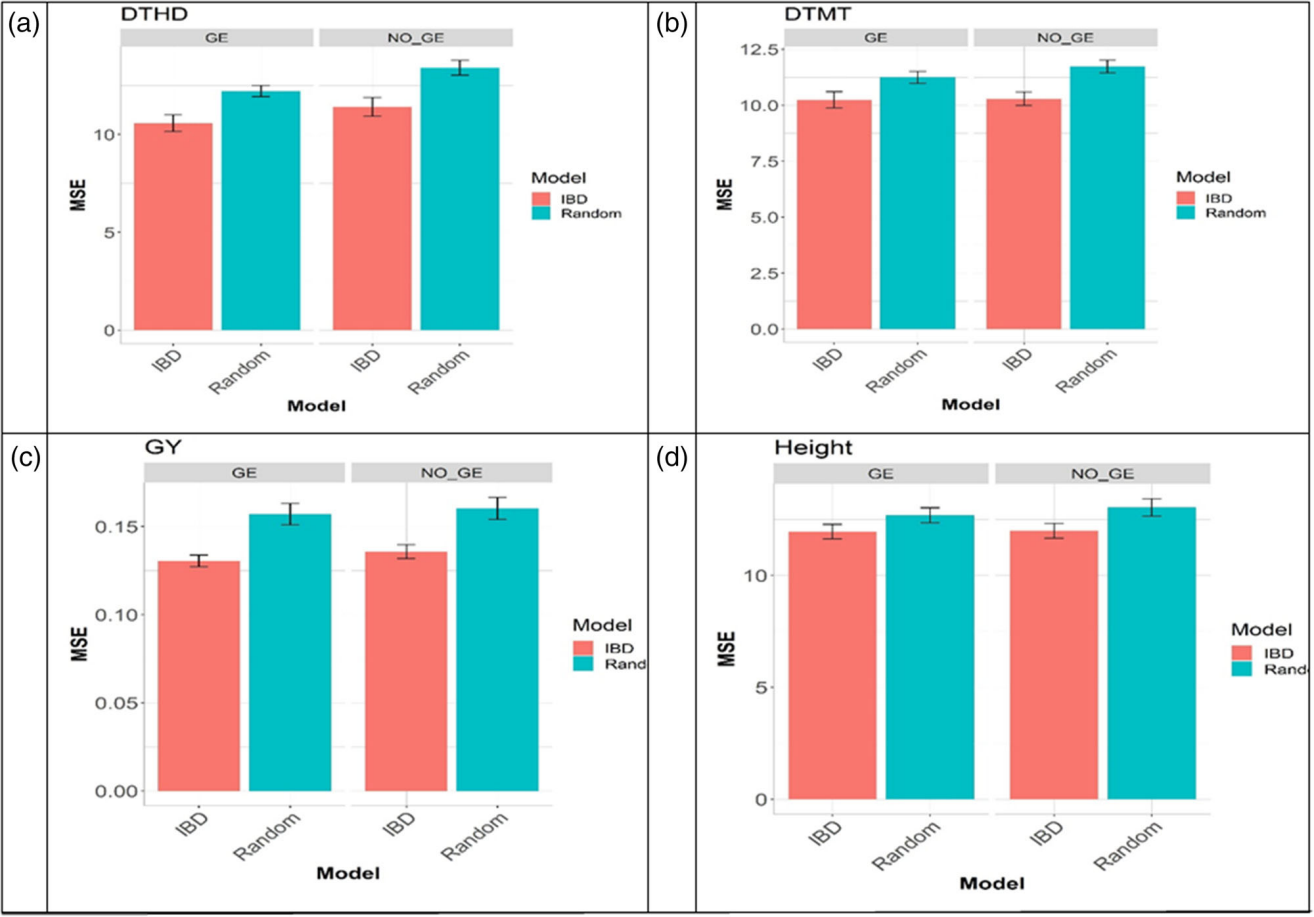
Data Set 1. Prediction performance in terms of mean square error (MSE) across locations for Data Set 1 (elite wheat yield trial years 2013–2014) for trait (a) days to heading (DTHD), (b) days to maturity (DTMT), (c) grain yield (GY), and (c) height. NO_GE, model ignores the genotype × location interaction; GE, model considers the genotype × location interaction; IBD, incomplete block designs

Next, we provide the results across locations including the G×E interaction for the four traits of Data Set 1. Across locations, we can observe that the best prediction performance (lower MSE) was obtained under the IBD allocation for the four traits DTHD, DTMT, GY, and height, where REs were 1.156 (15.6%), 1.098 (9.8%), 1.203 (20.3%), and 1.061(6.1%), respectively. These results indicate that the increase in prediction performance for IBD over the random allocation for traits DTHD, DTMT, GY, and height was 15.6, 9.8, 20.3, and 6.1% respectively (Figure 1, Table 5).

**TABLE 5:** Data Sets 1–5. Prediction performance in terms of mean square error (MSE) across locations for the five data sets under study

**Data**	**Interaction**	**Trait**	**MSE_IBD**	**SE_1**	**MSE_Random**	**SE_2**	**RE**
Data Set 1 (EYT)	GE	DTHD	10.563	0.217	12.210	0.141	1.156
		DTMT	10.236	0.186	11.244	0.133	1.098
		GY	0.131	0.002	0.157	0.003	1.203
		Height	11.954	0.166	12.679	0.169	1.061
	NO_GE	DTHD	11.411	0.244	13.407	0.193	1.175
		DTMT	10.290	0.153	11.728	0.147	1.140
		GY	0.136	0.002	0.160	0.003	1.180
		Height	11.986	0.169	13.030	0.198	1.087
Data Set 2 (EYT)	GE	DTHD	7.517	0.119	8.898	0.157	1.184
		DTMT	5.516	0.078	6.071	0.095	1.101
		GY	0.119	0.002	0.149	0.002	1.253
		Height	18.063	0.260	18.322	0.242	1.014
	NO_GE	DTHD	8.165	0.115	9.570	0.181	1.172
		DTMT	5.974	0.072	6.331	0.104	1.060
		GY	0.127	0.002	0.161	0.002	1.265
		Height	19.136	0.252	19.045	0.263	0.995
Data Set 3 Groundnut	GE	NPP	11.307	0.219	12.320	0.259	1.090
		PYPP	5.892	0.154	6.634	0.146	1.126
		SYPP	2.378	0.055	2.614	0.065	1.099
		YPH	206,096.445	4,465.824	229,646.854	6,114.155	1.114
	NO_GE	NPP	11.791	0.221	12.685	0.251	1.076
		PYPP	6.345	0.133	7.132	0.128	1.124
		SYPP	2.564	0.050	2.805	0.060	1.094
		YPH	223,512.394	4,731.489	247,620.162	6,274.576	1.108
Data Set 4 Wheat	GE	GY	0.453	0.008	0.527	0.011	1.164
	NO_GE		0.483	0.008	0.573	0.013	1.188
Data Set 5 Maize	GE	GY	0.1614	0.0019	0.1627	0.0028	1.008
	NO_GE		0.161	0.0021	0.1621	0.003	1.007

*Note*. DTHD, days to heading; DTMT, days to maturity; GE, model considers the genotype × location interaction; GY, grain yield; IBD, incomplete block design; MSE_IBD, MSE under IBD allocation; MSE_Random, MSE under random allocation; NO_GE, model ignores the genotype × location interaction; NPP, number of pods per plant; PYPP, pod yield per plant; RE, relative efficiency computed as the ratio of the MSE_IBD/MSE_Random; SE_1, SE of the MSE under the IBD allocation while ; SE_2, SE of the MSE under the random allocation; SYPP, seed yield per plant; YPH, yield per hectare.

When the G×E interaction was not considered (NO_GE), the best predictions were also observed under the IBD allocation with the following REs: 1.175 (DTHD), 1.140 (DTMT), 1.18 (GY), and 1.087 (height). This implies that the prediction performance of using the IBD over the random allocation for traits DTHD, DTMT, GY, and height increased by 17.5, 14.0, 18.0, and 8.07%, respectively (Figure 1, Table 5).

### 3.2 Data Set 2 (EYT years 2016–2017)

First, the prediction performance for each location is given including the G×E interaction. In Table 6 we can observe that the IBD allocation outperformed the random allocation in terms of MSE since for each of the traits, the relative efficiencies in most locations were >1. For trait DTHD, the REs observed were 1.107 (Bed5IR), 1.069 (EHT), 1.190 (Flat5IR), and 1.231 (FlatDrip), which means that the IBD outperformed the random allocation by 10.7, 6.9, 19.0, and 23.1%, respectively. For the DTMT trait, the IBD was more efficient than the random allocation by 15.8, 9.9, 15.4, and 6.2% in locations Bed5IR, EHT, Flat5IR, and FlatDrip, respectively, since the REs were 1.158, 1.099, 1.154, and 1.062, respectively (Table 6). For the GY trait, the IBD outperformed the random allocation by only 2.1, 1.6, and 0.7% in locations Bed5IR, EHT, and FlatDrip, respectively. Whereas for the height trait, the IBD was superior to the random allocation by 5.0% only in location Flat5IR.

**TABLE 6:** Data Set 2. Prediction performance in terms of mean square error (MSE) for each location for Data Set 2 (elite wheat yield trial [YET] years 2016–2017)

**Interaction**	**Trait**	**Location**	**MSE_IBD**	**SE_1**	**MSE_Random**	**SE_2**	**RE**
GE	DTHD	Bed5IR	10.161	0.122	11.251	0.237	1.107
		EHT	23.440	0.203	25.057	0.478	1.069
		Flat5IR	5.013	0.063	5.966	0.135	1.190
		FlatDrip	5.416	0.127	6.666	0.150	1.231
	DTMT	Bed5IR	6.527	0.108	7.558	0.228	1.158
		EHT	11.095	0.130	12.195	0.237	1.099
		Flat5IR	6.577	0.209	7.593	0.228	1.154
		FlatDrip	7.715	0.101	8.196	0.158	1.062
	GY	Bed5IR	0.179	0.002	0.183	0.003	1.021
		EHT	0.308	0.004	0.313	0.006	1.016
		Flat5IR	0.282	0.004	0.280	0.004	0.990
		FlatDrip	0.087	0.001	0.088	0.002	1.007
	Height	Bed5IR	22.544	0.237	22.214	0.390	0.985
		EHT	28.991	0.368	28.274	0.529	0.975
		Flat5IR	21.253	0.204	22.316	0.296	1.050
		FlatDrip	57.792	1.012	56.371	0.663	0.975
NO_GE	DTHD	Bed5IR	10.770	0.136	11.888	0.231	1.104
		EHT	25.560	0.187	26.886	0.574	1.052
		Flat5IR	5.918	0.074	6.792	0.137	1.148
		FlatDrip	6.148	0.143	7.433	0.144	1.209
	DTMT	Bed5IR	6.895	0.110	7.688	0.223	1.115
		EHT	11.719	0.148	12.449	0.238	1.062
		Flat5IR	6.947	0.204	7.688	0.207	1.107
		FlatDrip	9.306	0.139	9.914	0.213	1.065
	GY	Bed5IR	0.199	0.002	0.202	0.003	1.016
		EHT	0.321	0.005	0.327	0.005	1.019
		Flat5IR	0.316	0.006	0.314	0.004	0.991
		FlatDrip	0.112	0.002	0.113	0.003	1.007
	Height	Bed5IR	23.331	0.232	22.795	0.346	0.977
		EHT	30.896	0.495	29.829	0.562	0.965
		Flat5IR	21.087	0.159	21.936	0.236	1.040
		FlatDrip	65.608	1.067	64.024	0.623	0.976

*Note*. Bed5IR, bed planting with five irrigations; DTHD, days to heading; DTMT, days to maturity; EHT, early heat ; Flat5IR, flat planting with five irrigations; FlatDrip, flat planting with drip irrigation; GE, model considers the genotype × location interaction; GY, grain yield; IBD, incomplete block design; MSE_IBD, MSE under IBD allocation; MSE_Random, MSE under random allocation; NO_GE, model ignores the genotype × location interaction; RE, relative efficiency computed as the ratio of the MSE_IBD/MSE_Random; SE_1, SE of the MSE under the IBD allocation while ; SE_2, SE of the MSE under the random allocation.

Also, in Table 6, when ignoring the G×E interaction (NO_GE), we can observe in each location that the IBD allocation was better than the random allocation since for most of the traits, the RE in locations were >1. For the DTHD trait, the REs observed were 1.104 (Bed5IR), 1.052 (EHT), 1.148 (Flat5IR), and 1.209 (FlatDrip). Therefore, in this trait, the IBD allocation outperformed the random allocation by 10.4 (Bed5IR), 5.2 (EHT), 14.8 (Flat5IR), and 20.9% (FlatDrip) (Table 6). For the DTMT trait, the IBD outperformed the random allocation by 11.5 (Bed5IR, with RE = 1.115), 6.2 (EHT, with RE = 1.062), 10.7 (Flat5IR, with RE = 1.107), and 6.5% (FlatDrip, with RE = 1.065). For the GY trait, the IBD was better than the random allocation by 1.6, 1.9, and 0.7% in locations Bed5IR, EHT, and FlatDrip, respectively (Table 3). While in the height trait, the IBD outperformed the random allocation by only 4.0% only in location Flat5IR (with RE = 1.040) (Table 6).

**FIGURE 2:**
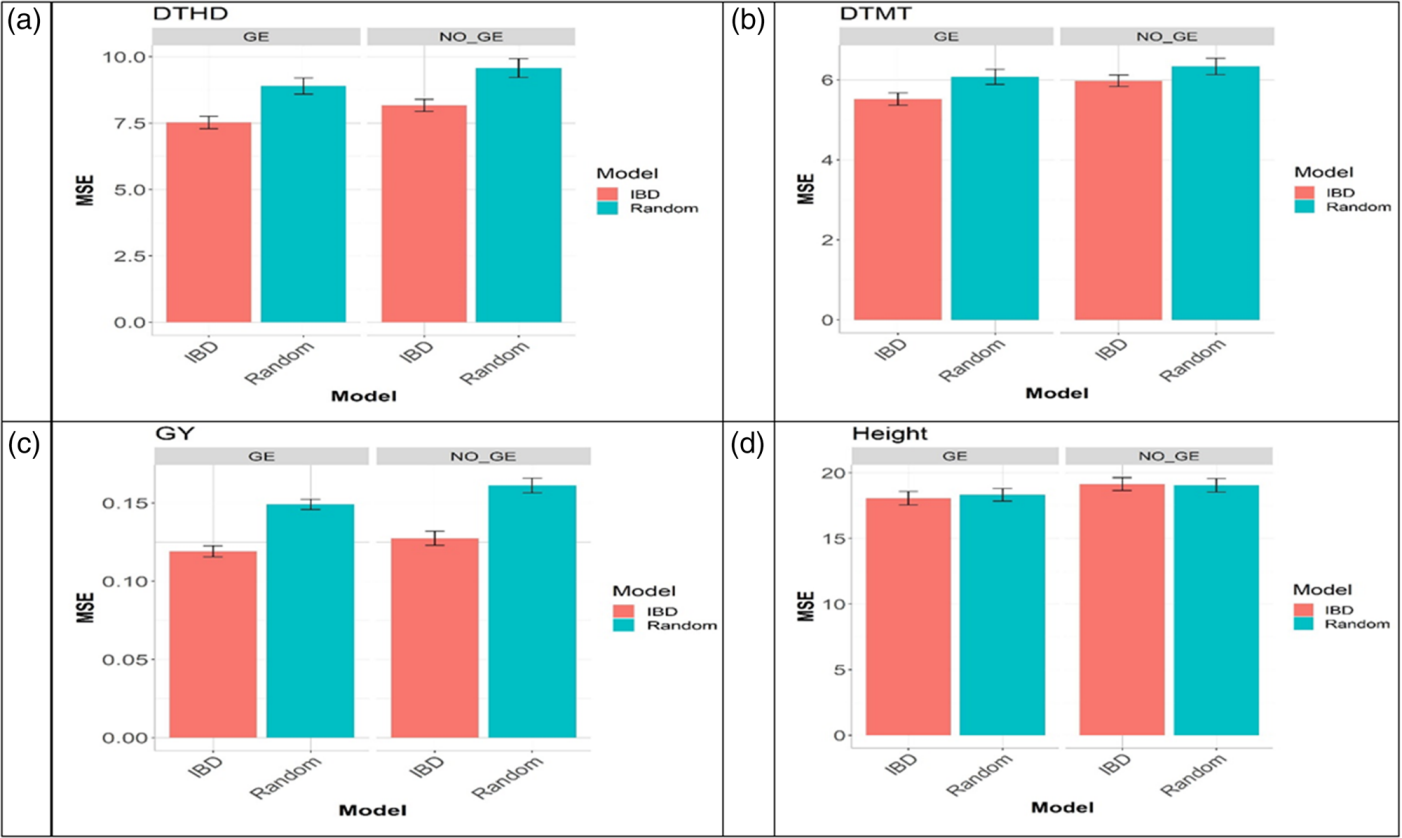
Data Set 2. Prediction performance in terms of mean square error (MSE) across locations for Data Set 2 (elite wheat yield trial years 2016–2017) for trait (a) days to heading (DTHD), (b) days to maturity (DTMT), (c) grain yield (GY), and (c) height. NO_GE, model ignores the genotype × location interaction; GE, model considers the genotype × location interaction; IBD, incomplete block designs

Across locations, including G×E interaction for the four traits of Data Set 2, we can observe that the best prediction performance (lower MSE) was obtained under the IBD allocation with the following REs in each trait: 1.184 (DTHD), 1.101 (DTMT), 1.253 (GY), and 1.014 (height). This means that the IBD increased prediction performance in terms of MSE over the random allocation by 18.4, 10.1, 25.3, and 1.4% in traits DTHD, DTMT, GY, and height, respectively (Figure 2, Table 5).

When ignoring the G×E interaction (NO_GE) across locations, the best predictions were also observed under the IBD allocation (Figure 2, Table 5) with the following relative efficiencies: 1.172 (DTHD), 1.060 (DTMT), 1.265 (GY), and 0.995 (height). This implies that the prediction performance of using the IBD over the random allocation increased in three out of the four traits DTHD, DTMT and GY by 17.2 (DTHD), 6.0 (DTMT), and 26.5% (GY) (Figure 2, Table 5).

### 3.3 Data Set 3 (groundnut)

**FIGURE 3:**
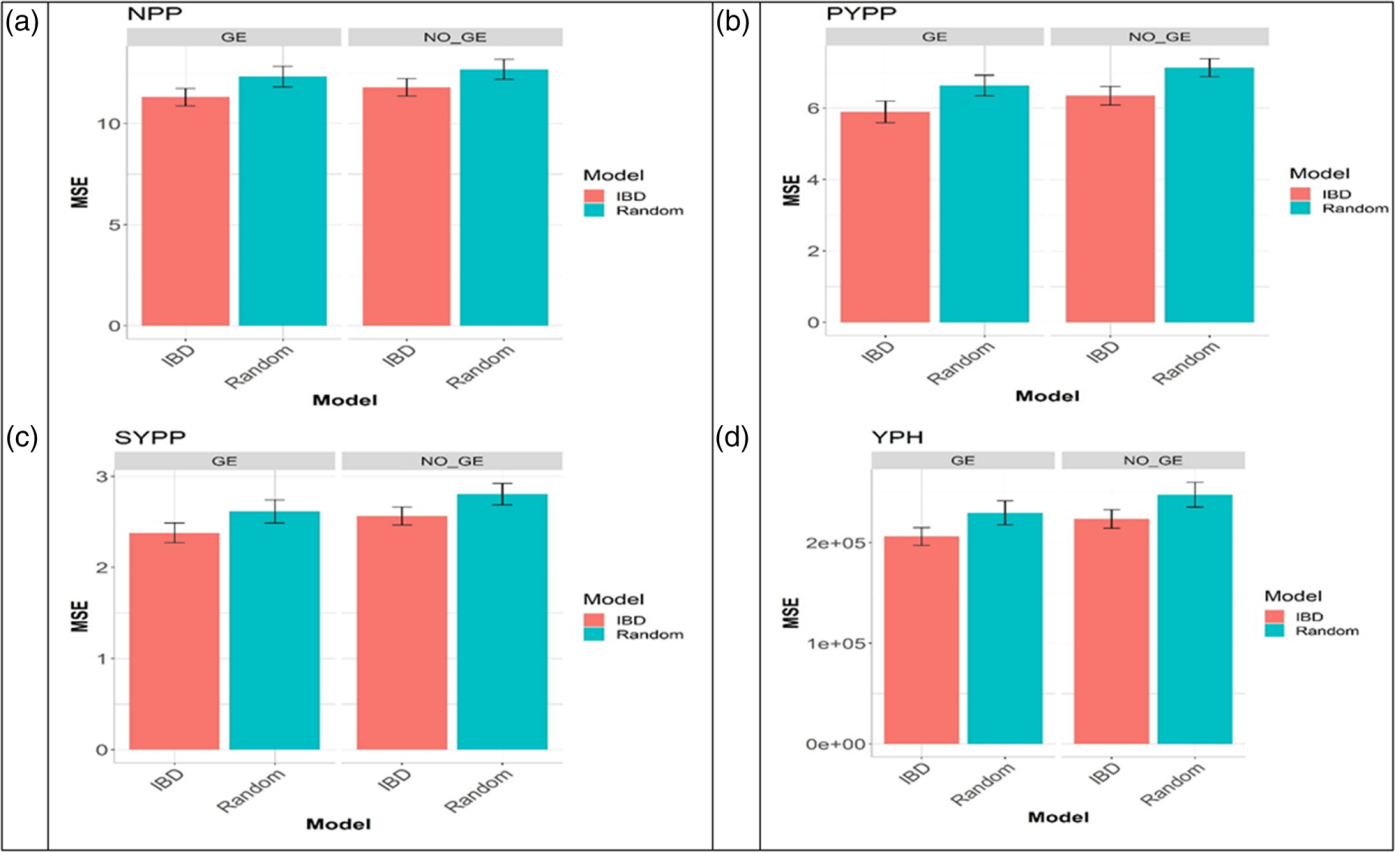
Data Set 3. Prediction performance in terms of mean square error (MSE) across locations for Data Set 3 (Groundnut) for trait (a) for number of pods per plant (NPP), (b) pod yield per plant (PYPP), (c) seed yield per plant (SYPP), and (d) yield per hectare (YPH). NO_GE, model ignores the genotype × location interaction; GE, model considers the genotype × location interaction; IBD, incomplete block designs

For this data set (groundnut), which also contained four traits (NPP, PYPP, SYPP, and YPH), we first provide the results including G×E interaction. Across locations, the IBD allocation outperformed the random allocation in terms of MSE since the REs obtained in the four traits are all >1: 1.090 (NPP), 1.126 (PYPP), 1.099 (SYPP), and 1.114 (YPH). This means that the increase in terms of prediction performance (lower MSE) of the IBD over the random allocation was of 9.0, 12.6, 9.9, and 11.4%, respectively (Figure 3, Table 5).

When the G×E interaction was not considered (NO_GE), the IBD allocation (Figure 3, Table 5) also outperformed the random allocation with the following relative efficiencies: 1.076 (NPP), 1.124 (PYPP), 1.094 (SYPP), and 1.108 (YPH). This implies that the prediction performance of using the IBD over the random allocation increased in the four traits by 7.6, 12.4, 9.4, and 10.8%, respectively (Figure 3, Table 5). Details of the prediction performance for each location for this data set can be found in Appendix Table B1.

### 3.4 Data Sets 4 (wheat) and 5 (maize)

**FIGURE 4:**
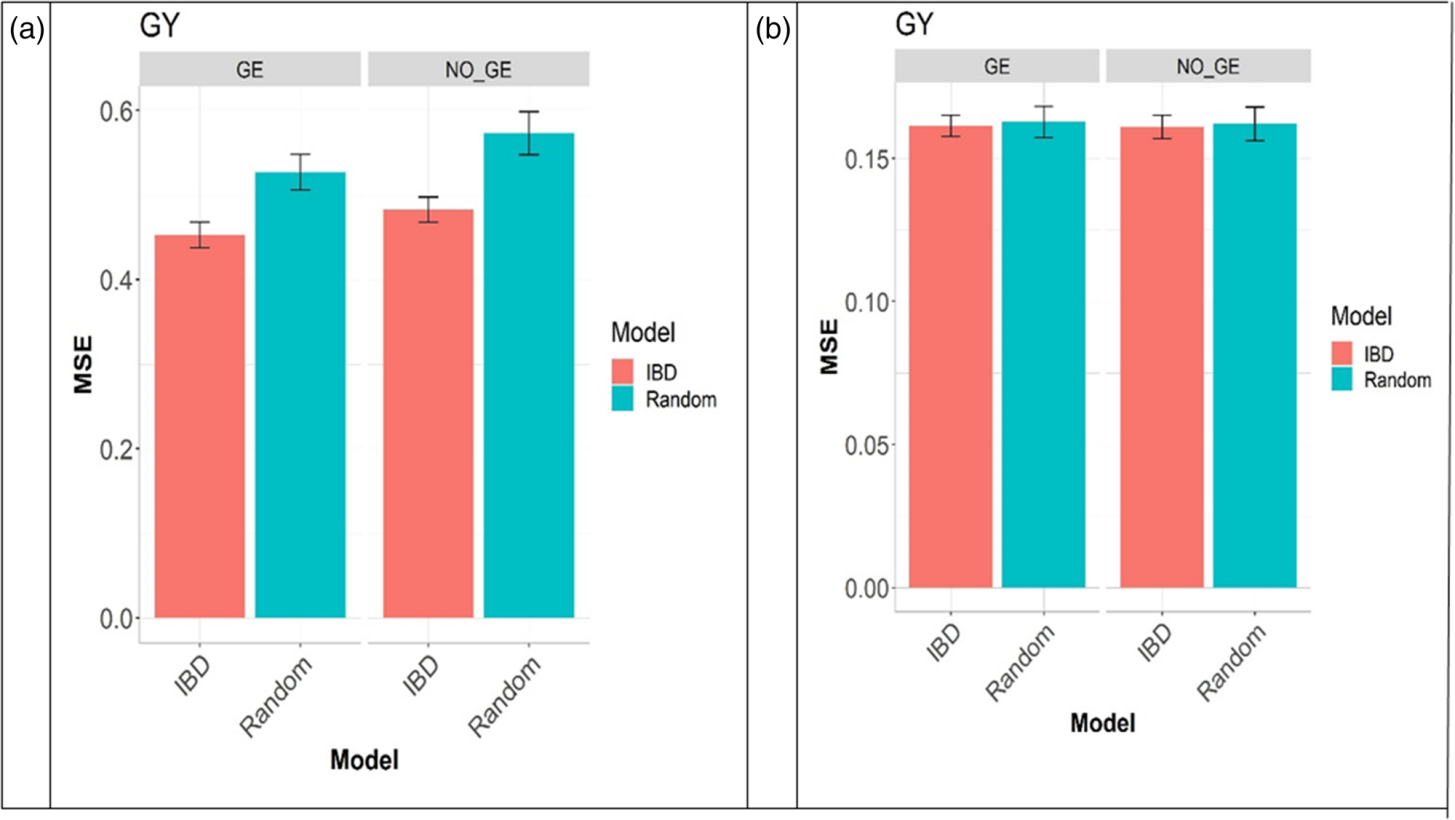
Data Sets 4 and 5. Prediction performance in terms of mean square error (MSE) across locations for Data Sets 4 and 5 for grain yield (GY) in (a) wheat data and (b) maize data. NO_GE, model ignores the genotype × location interaction; GE, model considers the genotype × location interaction

The wheat data set (Data Set 4) only contains the GY trait, and initial results include G×E interaction. Across locations, the IBD allocation outperformed the random allocation, in terms of MSE, by 16.4% (RE = 1.164) (Figure 4a, Table 5). When G×E interaction was ignored (NO_GE), the IBD allocation (Figure 4a) outperformed (RE = 1.188) the random allocation by 18.8% (Figure 4a, Table 5).

Similarly, the maize data set (Data Set 5) only contains the GY trait, and when considering the G×E interaction, we observed that the IBD allocation was superior to the random allocation by only 0.8% (RE = 1.008) in terms of MSE (Figure 4b, Table 5). When the G×E interaction was ignored (NO_GE), the IBD allocation (Figure 4b, Table 5) only had a 0.7% (RE = 1.007) gain over the random allocation (Figure 4b, Table 5). Details of the prediction performance for each location for these two data sets can be found in the Appendix Table B2 (Appendix).

## 4 DISCUSSION

Genomic selection can help optimize resources for the early selection of candidate genotypes. This is because only a sample of candidates need to be phenotyped and genotyped, while the remaining individuals must only be genotyped and use genome‐enabled prediction models to compute their genomic estimated breeding values. The accuracy of GS is linked to the quality of the predictions, and therefore, better predictions lead to more accurate GS methodology. For this reason, research to improve the efficiency of the GS methodology continues and our study aimed to test the use of IBDs for improving the efficiency of sparse testing. This has the aim of saving significant resources without a loss of prediction power compared with the standard practice of random allocation.

We found that the allocation of lines to locations (or environments) using IBD is superior to the random allocation across the data sets analyzed. In Data Set 1, IBD outperformed random allocation across locations and traits by between 6.1 and 20.3% (for GE) and between 8.07 and 18.0% for NO_GE. In Data Set 2 the IBD method outperformed the random method across locations and traits by between 1.4 and 18.4% (for GE) and by between 6 and 26.5% for NO_GE. In Data Set 3 across locations and traits, the IBD gain over the random method was between 9 and 12.6% for GE and between 7.6 and 12.4% for NO_GE. In Data Set 4, the IBD was superior to the random allocation method by 16.4% for GE and by 18.8% for NO_GE. These results also show that the superiority of the proposed IBD allocation is not significantly affected in its performance for the degree of G×E interaction, as exemplified by the five data sets studied. (Table 3). These results show empirical evidence that the allocation of lines to locations under the random allocation, which is common practice in plant breeding programs to design sparse testing in the context of genomic selection, is less efficient than the IBD allocation, which allocates the lines to locations under a classical experimental design called balanced IBD or partially balanced IBD.

However, the gain in predictive performance when using IBD over random allocation requires additional considerations. Specifically, the allocation of lines to locations using the IBD method is computationally more demanding than random allocation because the IBD allocation is built under a combinatorial process, which is considerably more time consuming. As the number of lines increases, so too does the time requirement for the allocation process. However, in real applications, this allocation process is only required once.

Additionally, the IBD allocation does not always guarantee that each line is allocated exactly to *r* out of *I* locations, meaning that the allocation is not always balanced. Even under these circumstances, the IBD allocation it expected to perform better overall than the random allocation. In this sense, it is of paramount importance to continue studying strategies for efficient sparse allocation of lines to locations to increase the efficiency of the GS. Our study presents new areas of opportunity to evaluate numerous IBDs. In addition to helping the optimization of parameter estimates, they can also be helpful for the construction of sparse testing allocation of lines to locations to increase prediction accuracy.

Experimental designs play an important role in plant breeding since appropriate experimental designs guarantee accurate data collection, proper data analysis, precise parameter estimates, and the right interpretation of the data (Masood et al., 2008). Additionally, breeders are aware that a properly planned experiment is necessary to ensure that the right type of data and a sufficient sample size and power are available to answer the research questions of interest as clearly and efficiently as possible.

In general, experimental designs are important in guaranteeing the quality of parameter estimates that provide more precision to the research questions at hand. Nevertheless, in the current study, we illustrated the use of experimental designs (partially balanced IBDs) for the sparse allocation of lines to locations, thus improving the accuracy of predictions. Therefore, from our results, we observe that the improvement of parameter estimates by using partially balanced IBDs for the sparse allocation of lines to locations also is translated to an increase of prediction performance.

The proposed allocation of lines to locations under partially balanced IBD (IBD allocation) is primarily of interest to breeders when their goal is to evaluate some lines (*J* denotes all lines available) in some locations such as evaluating each line in *r* out of *I* locations and making predictions of the untested (observed) lines in those locations. This allocation of lines to locations is done only with the goal of prediction, and the allocation of those lines in each location should be allocated to plots, blocks, and trials under a different and specific experimental design. From this local (inner experimental design) allocation of lines to plots, blocks, and trials, we can obtain the BLUEs using the specific experimental designs in each location. This means that it is possible within each location to allocate the lines under different experimental designs. Then with BLUEs of each line in each location, the model will be trained with the training set resulting from the allocation of lines to locations under the partially balanced IBD to predict the lines not observed in those locations.

Therefore, this process involves the use of two experimental designs: (a) one for the allocation of those lines to plots, blocks, and trials within each location (that can use a different experimental design in each location) and (b) another experimental design for building the training set with the BLUEs of the lines tested in each location. This second experimental design should be a partially balanced IBD that uses lines allocated for each location as the training set so that those unallocated lines to each location as the testing set will be predicted with the trained model. Our proposed approach coincides with what is called two‐phase experimental design, where a randomization in each phase is performed to be able to obtain robust phenotypic data (McIntyre, 1955). This approach has been proposed in the context of plant breeding for improving parameter estimates in horseshoe pelargonium [*Pelargonium zonale* (L.) LʼHér.] (Brien et al., 2011; Molenaar et al., 2018, 2017); however, to the best of our knowledge this is the first time that this two‐phase experimental design is proposed for the context of genome‐based selection.

In this study, we do not evaluate the role of population structure on the proposed method. This ceased to be a concern when de los Campos et al. (2015) pointed out that population structure does not play the role of a confounding factor, rather a modified factor. However, for a complete understanding of these issues, future studies should be conducted to be able to quantify how the population structure of the genomic relationship matrix or kinship matrix affects the prediction performance of the incomplete blocks created to implement the sparse testing method proposed here.

Furthermore, more evaluations are necessary since even though the five data sets are from three different crops and with different levels of explained total variability of each of its variance components, they are not representative of all crops and variability of data generated in plant breeding programs. Finally, as pointed out above, it is possible that other forms of experimental IBDs can be used to design sparse testing methods for allocating lines to locations. This will further support the goal of increasing the prediction performance in the context of GS. However, specific additional designs still need to be evaluated to ensure that they help increase the prediction performance.

## 5 CONCLUSION

In this study, we proposed the use of IBDs for sparse testing allocation of lines to locations for genomic prediction. We found that the proposed IBD allocation helps to significantly improve predictions compared with the standard random allocation of lines to locations. However, we also found that when the data set is larger, the allocation of lines using IBDs are more time consuming and computationally intensive. However, this component is unlikely to be a major barrier, as the allocation is only required once in a breeding application. The proposed IBD method contributes to increasing the availability of sparse testing methods for plant breeding that makes the GS methodology more efficient, as it provides better prediction performance than the random allocation of lines to locations. However, we suggest performing more empirical evaluations to accumulate further evidence of the utility of IBD for an efficient allocation of lines to locations for sparse testing in GS. Other experimental designs can be evaluated for their use in sparse testing genomic prediction, supporting an increase in the power of the GS methodology.

## AUTHOR CONTRIBUTIONS

Jose Crossa: Conceptualization; Investigation. Osval Montesinos‐Lopez: Conceptualization; Investigation; Methodology; Formal analysis; Writing. Ricardo Acosta: Conceptualization; Data curation; Formal analysis. Rajeev K. Varshney: Resources; Writing – review & editing. Alison Bentley: Writing – review & editing.

## CONFLICT OF INTEREST

The authors declare no conflict of interest.

## Figures and Tables

**FIGURE A1:**
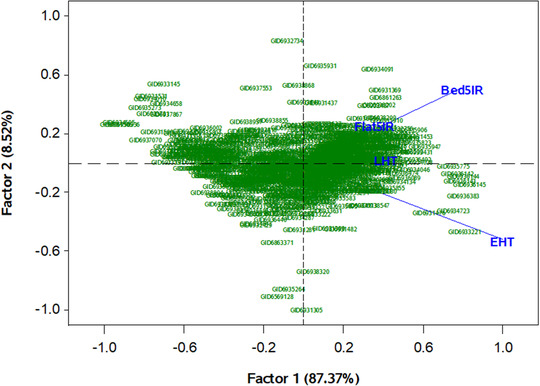
Biplot for trait days to heading (DTHD) of Data Set 1 (elite wheat yield trial)

**FIGURE A2:**
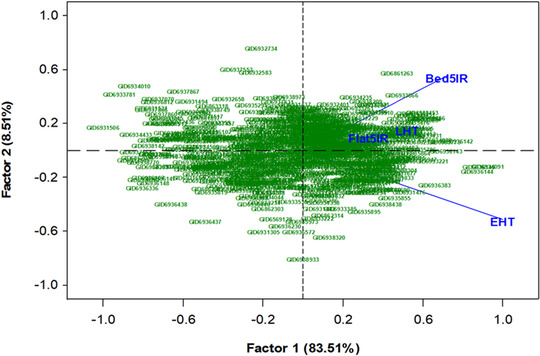
Biplot for trait days to maturity (DTMT) of Data Set 1 (elite wheat yield trial)

**FIGURE A3:**
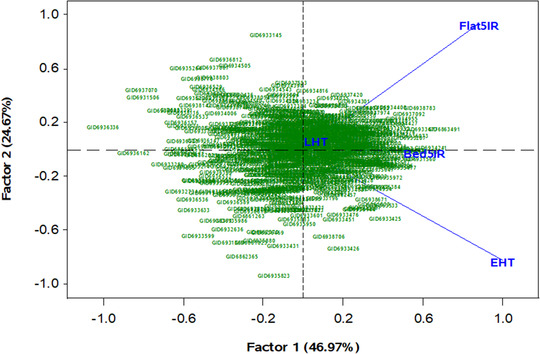
Biplot for trait grain yield (GY) of Data Set 1 (elite wheat yield trial)

**FIGURE A4:**
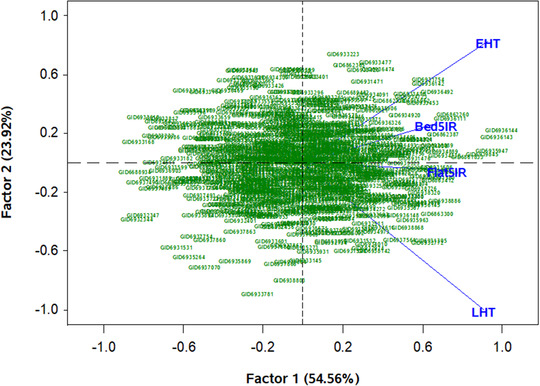
Biplot for trait height of Data Set 1 (elite wheat yield trial)

**FIGURE A5:**
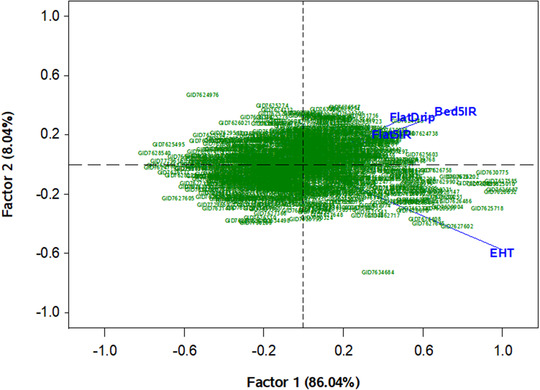
Biplot for trait days to heading (DTHD) of Data Set 2 (elite wheat yield trial)

**FIGURE A6:**
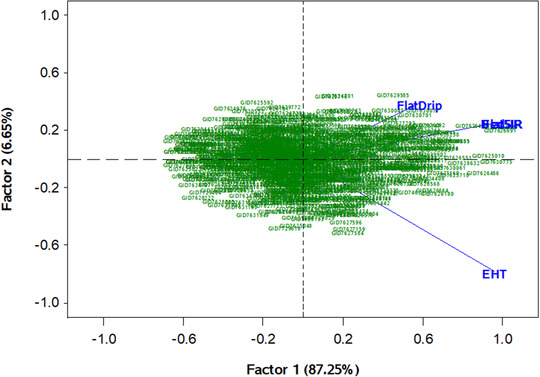
Biplot for trait days to maturity (DTMT) of Data Set 2 (elite wheat yield trial)

**FIGURE A7:**
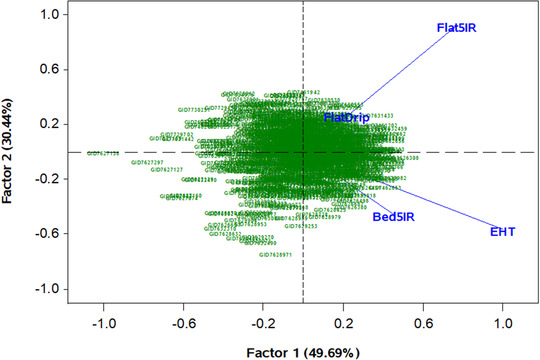
Biplot for trait grain yield (GY) of Data Set 2 (elite wheat yield trial)

**FIGURE A8:**
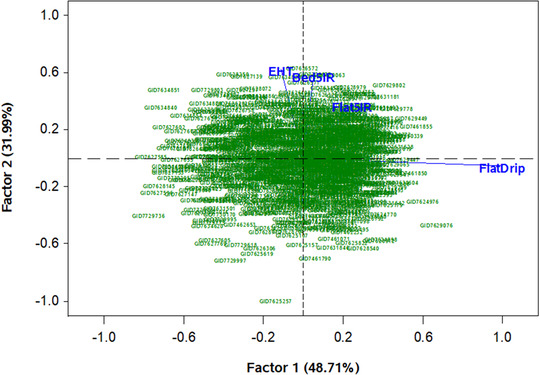
Biplot for trait height of Data Set 2 (elite wheat yield trial)

**FIGURE A9:**
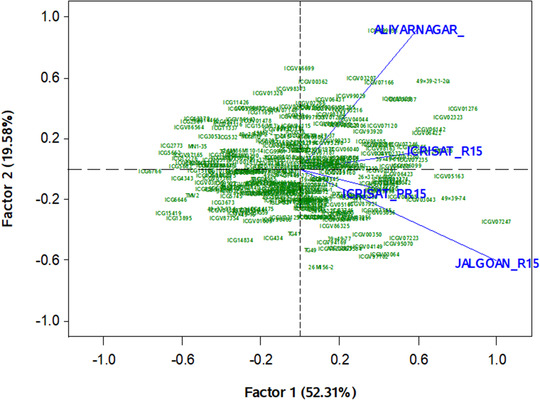
Biplot for trait number of pods per plant (NPP) of groundnut data set

**FIGURE A10:**
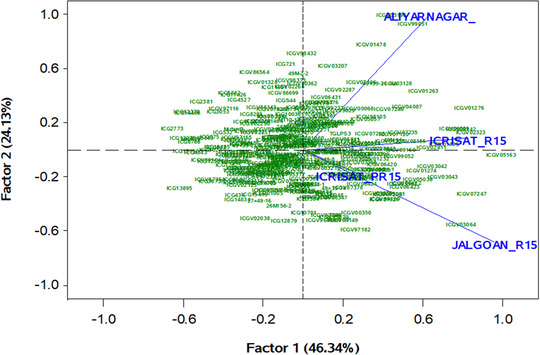
Biplot for trait pod yield per plant (PYPP) of groundnut data set

**FIGURE A11:**
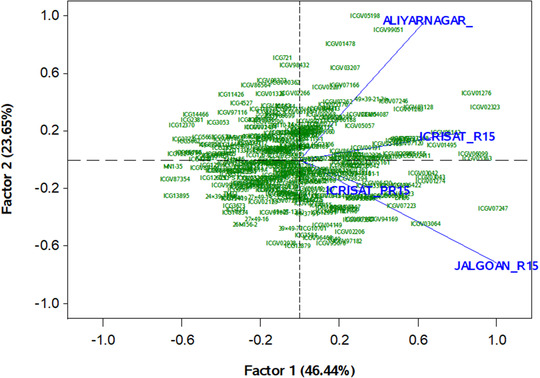
Biplot for trait seed yield per plant (SYPP) of groundnut data set

**FIGURE A12:**
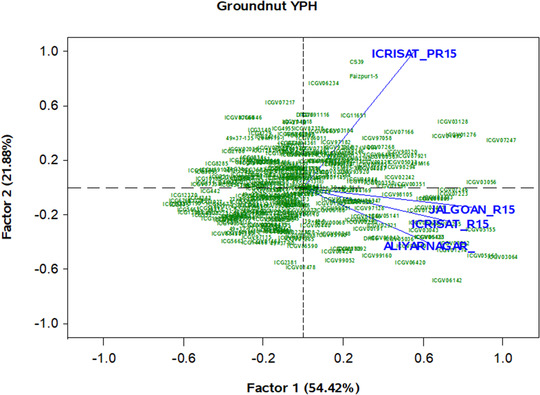
Biplot for trait yield per hectare (YPH) of groundnut data set

**FIGURE A13:**
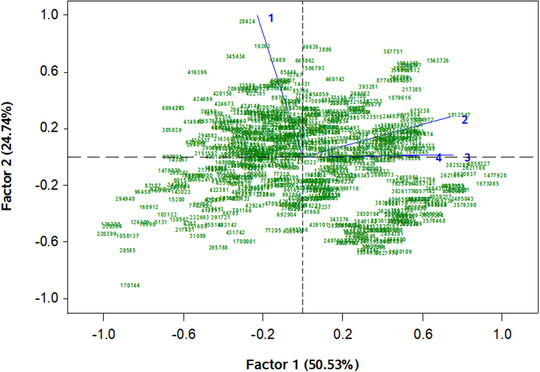
Biplot for trait grain yield (GY) of wheat data set

**FIGURE A14:**
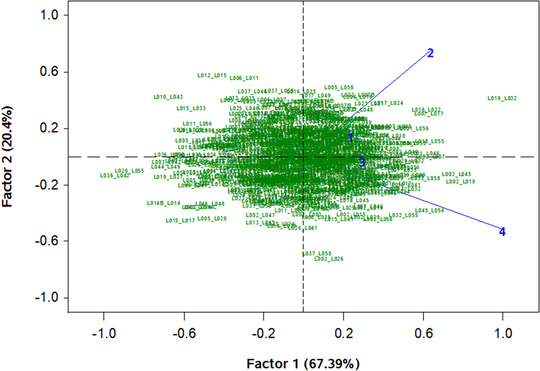
Biplot for trait grain yield (GY) of maize data set

## Data Availability

Phenotypic and marker data, as well the R code for computing the training–testing sets under the random and IBD allocations used in this study, are available online (https://github.com/osval78/sparse_testing).

## APPENDIX A

### A.1

The site regression model (SREG) (Crossa & Cornelius, 1997) provides the multiplicative operators computed from a reduced‐rank model matrix of deviations of the parametric cell mean of the genotype (G) in the environment (E) from the mean of the environment (i.e., the effects of the genotypes plus the effect of the G×E).

For the SREG biplot [Figures A1, A2, A3, A4, A5, A6, A7, A8, A9, A10, A11, A12, A13, A14, where cultivars are in green color and environments (or sites) are in blue colors], the cosine of the angle between two cultivar (or environment) vectors approximates the correlation between the cultivars (or environments) with respect to the main effect of cultivar plus the G×E. Acute angles indicate positive correlation, with parallel vectors (in exactly the same directions) representing a correlation of 1.0. Obtuse angles represent negative correlations, with opposite directions indicating a correlation of −1.0. Perpendicularity of directions indicates a correlation of zero. Environmental vectors having the same direction as the cultivar vectors have positive cultivar plus the G×E effects (that is, these environments favored these cultivars), whereas vectors in the opposite direction have negative cultivar plus G×E. Thus, environmental vectors on the same direction indicate a less complex G×E than those located in opposite directions. For example, biplot of Figure A13 for grain yield (GY) of data set wheat displayed Site 1 as being on different direction than Sites 2, 3, and 4, thus showing a more complex G×E than that of Figure A14 for GY of data set maize, where all sites pointed towards the right‐hand side of the biplot.

## APPENDIX B

### B.1

**TABLE B1:** Data Set 3. Prediction performance in terms of mean square error (MSE) for each location for Data Set 3 (groundnut)

**Interaction**	**Trait**	**Location**	**MSE_IBD**	**SE_1**	**MSE_Random**	**SE_2**	**RE**
GE	NPP	ALIYARNAGAR_R15	22.473	0.614	21.781	0.526	0.969
		ICRISAT_PR15‐16	14.782	0.270	15.543	0.376	1.052
		ICRISAT_R15	16.936	0.461	17.278	0.434	1.020
		JALGOAN_R15	24.602	0.521	26.006	0.641	1.057
	PYPP	ALIYARNAGAR_R15	13.216	0.444	13.929	0.554	1.054
		ICRISAT_PR15‐16	7.520	0.278	7.632	0.325	1.015
		ICRISAT_R15	9.162	0.277	9.356	0.204	1.021
		JALGOAN_R15	12.766	0.396	13.336	0.365	1.045
	SYPP	ALIYARNAGAR_R15	5.149	0.179	5.342	0.187	1.038
		ICRISAT_PR15‐16	3.118	0.096	3.121	0.134	1.001
		ICRISAT_R15	3.540	0.085	3.696	0.085	1.044
		JALGOAN_R15	5.124	0.181	5.329	0.138	1.040
	YPH	ALIYARNAGAR_R15	321,754.139	8,464.719	312,837.826	14,259.624	0.972
		ICRISAT_PR15‐16	478,758.632	17,336.600	485,554.901	19,143.458	1.014
		ICRISAT_R15	233,630.120	5,897.236	237,905.723	6,313.021	1.018
		JALGOAN_R15	372,801.784	11,850.626	384,906.854	21,458.950	1.032
NO_GE	NPP	ALIYARNAGAR_R15	23.125	0.587	22.392	0.440	0.968
		ICRISAT_PR15‐16	15.943	0.323	16.799	0.446	1.054
		ICRISAT_R15	17.220	0.478	17.445	0.397	1.013
		JALGOAN_R15	25.445	0.505	26.407	0.641	1.038
	PYPP	ALIYARNAGAR_R15	13.545	0.503	14.309	0.517	1.056
		ICRISAT_PR15‐16	8.570	0.337	8.844	0.350	1.032
		ICRISAT_R15	10.220	0.300	10.386	0.165	1.016
		JALGOAN_R15	13.743	0.330	14.083	0.361	1.025
	SYPP	ALIYARNAGAR_R15	5.312	0.208	5.511	0.172	1.037
		ICRISAT_PR15‐16	3.507	0.128	3.583	0.143	1.022
		ICRISAT_R15	3.922	0.105	4.042	0.089	1.031
		JALGOAN_R15	5.541	0.151	5.606	0.128	1.012
	YPH	ALIYARNAGAR_R15	331,125.618	12,951.686	325,784.803	15,175.139	0.984
		ICRISAT_PR15‐16	536,828.439	19,544.274	550,281.314	19,245.926	1.025
		ICRISAT_R15	259,150.933	7,038.008	259,444.711	8,705.813	1.001
		JALGOAN_R15	400,139.360	16,427.775	410,414.464	23,356.135	1.026

*Note*. GE, model considers the genotype × location interaction; GY, grain yield; IBD, incomplete block design; MSE_IBD, MSE under IBD allocation; MSE_Random, MSE under random allocation; NO_GE, model ignores the genotype × location interaction; NPP, number of pods per plant; PYPP, pod yield per plant; RE, relative efficiency computed as the ratio of the MSE_IBD/MSE_Random; SE_1, SE of the MSE under the IBD allocation while ; SE_2, SE of the MSE under the random allocation; SYPP, seed yield per plant; YPH, yield per hectare.

**TABLE B2:** Data Sets 4 (wheat) and 5 (maize). Prediction performance in terms of mean square error (MSE) for each location for Data Sets 4 and 5 (wheat and maize) for trait grain yield (GY)

**Data**	**Interaction**	**Location**	**MSE_IBD**	**SE_1**	**MSE_Random**	**SE_2**	**RE**
Wheat	GE	1	0.802	0.016	0.828	0.016	1.033
		2	0.757	0.012	0.744	0.014	0.983
		3	0.829	0.015	0.828	0.014	0.999
		4	0.808	0.016	0.783	0.016	0.969
	NO_GE	1	1.109	0.020	1.110	0.019	1.001
		2	0.796	0.014	0.799	0.014	1.003
		3	0.815	0.018	0.822	0.015	1.009
		4	0.865	0.013	0.841	0.013	0.973
Maize	GE	1	0.076	0.002	0.080	0.002	1.054
		2	0.309	0.005	0.331	0.006	1.071
		3	0.112	0.002	0.112	0.001	1.002
		4	0.482	0.008	0.472	0.010	0.980
	NO_GE	1	0.082	0.002	0.087	0.002	1.064
		2	0.311	0.005	0.332	0.007	1.066
		3	0.114	0.002	0.115	0.001	1.003
		4	0.496	0.008	0.486	0.011	0.981

*Note*. GE, model considers the genotype × location interaction; IBD, incomplete block design; MSE_IBD, MSE under IBD allocation; MSE_Random, MSE under random allocation; NO_GE, model ignores the genotype × location interaction; RE, relative efficiency computed as the ratio of the MSE_IBD/MSE_Random; SE_1, SE of the MSE under the IBD allocation while ; SE_2, SE of the MSE under the random allocation.
